# The relation between hypoxia and proliferation biomarkers with radiosensitivity in locally advanced laryngeal cancer

**DOI:** 10.1007/s00405-023-07951-9

**Published:** 2023-04-08

**Authors:** Karlijn van den Bovenkamp, Bert van der Vegt, Gyorgy B. Halmos, Lorian Slagter-Menkema, Johannes A. Langendijk, Boukje A. C. van Dijk, Ed Schuuring, Bernard F. A. M. van der Laan

**Affiliations:** 1 Department of Otorhinolaryngology/Head and Neck Surgery, University Medical Center Groningen, University of Groningen, Groningen, The Netherlands; 2 Department of Pathology, University Medical Center Groningen, University of Groningen, Hanzeplein 1, 9723 GZ Groningen, The Netherlands; 3 Department of Radiotherapy, University Medical Center Groningen, University of Groningen, Groningen, The Netherlands; 4 Department of Epidemiology, University Medical Center Groningen, University of Groningen, Groningen, The Netherlands; 5 Department of Research and Development, Netherlands Comprehensive Cancer Organisation (IKNL), Utrecht, The Netherlands; 6 Present Address: Department of Otorhinolaryngology/Head and Neck Surgery, Haaglanden Medical Center, The Hague, The Netherlands

**Keywords:** Laryngeal squamous cell carcinoma, Radiosensitivity, Hypoxia, Proliferation, Tumour markers

## Abstract

**Purpose:**

Treatment decision-making in advanced-stage laryngeal squamous cell carcinoma (LSCC) is difficult due to the high recurrence rates and the desire to preserve laryngeal functions. New predictive markers for radiosensitivity are needed to facilitate treatment choices. In early stage glottic LSCC treated with primary radiotherapy, expression of hypoxia (HIF-1α and CA-IX) and proliferation (Ki-67) tumour markers showed prognostic value for local control. The objective of this study is to examine the prognostic value of tumour markers for hypoxia and proliferation on locoregional recurrent disease and disease-specific mortality in a well-defined cohort of patients with locally advanced LSCC treated with primary, curatively intended radiotherapy.

**Methods:**

In pre-treatment biopsy tissues from a homogeneous cohort of 61 patients with advanced stage (T3–T4, M0) LSCC primarily treated with radiotherapy, expression of HIF-1α, CA-IX and Ki-67 was evaluated with immunohistochemistry. Demographic data (age and sex) and clinical data (T- and N-status) were retrospectively collected from the medical records. Cox regression analysis was performed to assess the relation between marker expression, demographic and clinical data, and locoregional recurrence and disease-specific mortality.

**Results:**

Patients with high expression of HIF-1α developed significantly more often a locoregional recurrence (39%) compared to patients with a low expression (21%) (*p* = 0.002). The expression of CA-IX and Ki-67 showed no association with locoregional recurrent disease. HIF-1α, CA-IX and Ki-67 were not significantly related to disease-specific mortality. Clinical N-status was an independent predictor of recurrent disease (*p* < 0.001) and disease-specific mortality (*p* = 0.003). Age, sex and T-status were not related to locoregional recurrent disease or disease-specific mortality.

**Conclusion:**

HIF-1α overexpression and the presence of regional lymph node metastases at diagnosis were independent predictors of locoregional recurrent disease after primary treatment with curatively intended radiotherapy in patients with locally advanced LSCC.

## Introduction

The treatment of advanced-stage laryngeal cancer is challenging. This is not only due to the high recurrence rates but also because of the desire to preserve essential laryngeal functions such as speech and swallow. Based on two studies, the standard treatment of advanced laryngeal squamous cell carcinoma (LSCC) changed, favouring primary (chemo)radiotherapy over total laryngectomy resulting in a better functional outcome [1–3]. Unfortunately, the survival of laryngeal cancer did not improve in the last decades despite many technical improvements. The reasons for this stagnation are not fully understood, but there are concerns about the influence of the shift towards non-surgical treatment on survival [4, 5].

Survival is associated with the occurrence of recurrences, with the worst prognosis in the case of locoregional failure within the first year after treatment [6]. Data on recurrence rates for the different treatment strategies are controversial [7–10]. However, when residual or recurrent disease occur after radiotherapy, salvage therapy is not always possible and has inferior oncological and functional outcome compared to primary surgery [11]. Today, only clinical characteristics, such as the TNM classification, are used to predict response to radiotherapy. But although these features perform reasonably well in large patient groups, they are unable to accurately predict response in an individual patient. Therefore, to improve treatment decision-making in advanced-stage LSCC, there is a need for new predictive markers.

Recently, a systematic review of tumour markers was performed, investigating their ability to predict local control in patients with LSCC treated with definitive radiotherapy [12]. Fifty-two studies reporting on 118 biomarkers were selected. Most studies selected patients with early-stage LSCC, and only a few included also advanced stage disease; however, none of the studies consisted of a homogeneous group of advanced-stage LSCC [12]. EGFR and P53 showed consistent evidence for not being predictive of local control after primary radiotherapy, whereas the proliferation marker Ki-67 is of interest for predicting local control in laryngeal cancer patients treated with radiotherapy. Other clusters of markers (for instance markers involved in cell cycle, angiogenesis and hypoxia) showed no consistent evidence towards being predictors of local control. Large diversity in research methods between studies and the lack of standardization was the main explanation for the contradictory outcomes. When using patients with a T1–T2 LSCC in the glottis only, we previously demonstrated that hypoxia biomarkers HIF-1α and CA-IX were of prognostic value for local control after primary radiotherapy [13]. Interestingly, no significant association between these markers and local control was found for early-stage supraglottic LSCC [14].

As assessing radiosensitivity in advanced-stage laryngeal cancer may have a significant impact on treatment policy in advanced stage laryngeal cancer, the aim of this study was to assess the prognostic value of tumour markers for hypoxia (HIF-1α and CA-IX) and proliferation (Ki-67) in a well-defined consecutive series of patients with advanced stage (T3–T4, M0) laryngeal squamous cell carcinoma treated with primary, curatively intended radiotherapy with regards to locoregional recurrent disease and disease-specific mortality.

## Materials and methods

### Patients

Patients treated for LSCC at the University Medical Center Groningen were included in the Netherlands Cancer Registry (NCR)-database and in the histopathology and cytopathology network and archive in het Netherlands (PALGA). From these databases, we selected patients that met the following inclusion criteria: (1) diagnosis between 1990 and 2014, (2) primary cT3 or cT4 biopsy-proven LSCC without distant metastases, (3) primarily curatively treated with radiotherapy only, and (4) sufficient pre-treatment, formalin-fixed, and paraffin-embedded biopsy tissue available in the biobank at the Department of Pathology of our institution.

Patients with previous irradiation of the head and neck region were excluded. Clinical, histopathological, and follow-up data were retrospectively collected from the medical records.

Pre-treatment biopsy material was collected for these patients. All formalin-fixed, paraffin-embedded pre-treatment biopsy slides were revised by a dedicated head and neck pathologist (BvdV). Pre-treatment biopsy was part of the standard clinical diagnostic process, and was handled following the Dutch ‘Codes of conduct’ [15]. The Local ethics Review Board Pathology non-WMO studies (LTc Pathology) of the University Medical Centre Groningen, approved this study to be outside the scope of the Medical Research Involving Human Subjects Act (WMO) (registration number: 202100893).

### Treatment and follow-up

Treatment consisted of curatively intended primary radiotherapy using conventional three-dimensional conformal radiotherapy (3D-CRT) and since 2007 intensity-modulated radiotherapy (IMRT). 3D-CRT and IMRT have comparable oncological long-term outcome with less toxicity after IMRT [16].

3D-CRT was given in 23 × 2 Gy fractions to the elective neck levels, with a sequential boost on the primary tumour and lymph node metastases to a total dose of 70 Gy. For patients treated with IMRT, a simultaneous integrated boost technique was used. Most patients received bilateral elective irradiation of the neck in 35 × 1.55 Gy fractions, while the primary tumour and lymph node metastases received a total dose of 70 Gy in 2 Gy fractions.

Follow-up after completing RT was in accordance with the Dutch Head and Neck Society (NWHHT) guidelines [17] and consisted of flexible laryngoscopy and physical examination of the neck every 3 months during the first 2 years and every 6 months thereafter up to 5 years after treatment. Approximately 8 weeks after completion of radiotherapy CT imaging of the head and neck region was performed for treatment response.

### Immunohistochemistry and TMA construction

3 μm sections were cut from the tumour paraffin blocks using a standard microtome, and haematoxylin and eosin staining was performed to verify histology. A tissue microarray (TMA) was constructed as previously described [18], and haematoxylin and eosin staining was performed to confirm the quality of the TMA and the presence of tumour cells in each core. In case no tumour was found in the TMA, 3 μm sections of the original tissue block were used for immunohistochemistry. All TMA contained a set of seven different normal tissues as control. The slides were deparaffinised using xylene twice for 10 min and rehydrated through a series of decreasing ethanol dilutions and phosphate-buffered saline (PBS). To block endogenous peroxidase activity, 0.3% hydrogen peroxide was applied for 30 min at room temperature.

### Immunohistochemical staining and scoring of HIF-1α and CA-IX

HIF-1α expression was detected using the murine monoclonal antibody Clone 54 according to the manufacturers’ instructions (BD Biosciences, Franklin Lakes, NJ) as we reported previously [13]. For antigen retrieval tissue slides were heated in a microwave in preheated Tris/ethylene-diamine-tetra-acetic acid buffer (pH 9.0) for 15 min at 100 °C. CA-IX expression protein was detected using the murine monoclonal antibody M75 (provided by Jaromir Pastorek, University of Bratislava, Slovakia) without antigen retrieval as described earlier [13]. 0.3% hydrogen peroxide was applied for 30 min at room temperature to block endogenous peroxidase activity. The slides were incubated for 1 h with the CA-IX and overnight at 4 °C with the HIF-1α antibody. Secondary antibodies, Rabbit anti-Mouse Biotin (CA-IX, dilution 1:300) and Rabbit anti-Mouse HRP (HIF-1α, dilution 1:100) (Dakocytomation), were diluted in 1% bovine serum albumin/PBS complemented with 1% human AB serum and applied for 30 min at room temperature. Tertiary antibody Streptavidine HRP (CA-IX) and Goat anti-Rabbit HRP (HIF-1α) 1:100 (Dakocytomation) was applied for 30 min at room temperature. The peroxidase reaction was performed by applying 3,30-diaminobenzide tetrachloride for 10 min. After washing with water, the slides were counterstained for 2 min with hematoxylin and mounted.

All TMA cores were evaluated by two observers (KB, LS-M) separately, both of which were blinded for the clinical outcomes, using scoring criteria as previously described [13, 14]. Differences in the scores were discussed and resolved at a conference microscope. The percentage of positive tumour cells was scored for each antibody, using only nuclear staining for HIF-1α, and only membranous staining for CA-IX. Cut-off values of the percentages for dichotomisation of the data were in accordance with the cut-off values used in previous studies performed in our institution [13, 14], and were 0.5% for HIF-1α, 12.5% for CA-IX.

### Automatic immunostaining and digital automatic scoring of Ki-67

Ki-67 expression was evaluated as reported recently [19] with CONFIRM^®^ anti-Ki-67 [30-9] Rabbit Monoclonal Primary Antibody (Ventana Medical Systems, Illkirch Cedex, France) using the automated Ventana BenchMark Ultra immunostainer (Ventana Medical Systems). Antibodies were pre-diluted by the manufacturer and staining was performed according to the manufacturer’s recommendations and protocol. Antigen retrieval time was 36 min using Cell Conditioning 1, pH 9 (Ventana Medical Systems). Antibody incubation time was 28 min.

A Philips Ultra Fast Scanner 1.6 (Philips, Eindhoven, The Netherlands) was used to digitize the stained slides as described before [20]. After manual annotation of the tumour tissue, the percentage of nuclear staining of Ki-67 was automatically analysed with a nuclear classification algorithm using Visiopharm Integrator System (VIS) platform (version 6.9.0.2779, Visiopharm, Hørsholm, Denmark) [20]. The cut-off value of the percentages for dichotomization of the data was in concordance with the cut-off value previously used in our institution [19], and was 50%.

### Statistical analysis

For survival analyses, age was dichotomized into < 65 years and ≥ 65 years, and N-status was dichotomized into N0 (no regional lymph node metastases) and N+ (presence of regional lymph node metastases). Locoregional recurrence was defined as tumour recurrence at the primary tumour site or cervical lymph nodes within 2 years from the date of completion of radiotherapy. Time calculations were performed using the date of completion of radiotherapy as starting point and the day of locoregional recurrence, death, or last follow-up as an endpoint. Disease-specific mortality was defined as death as a consequence of LSCC within 5 years from the date of completion of radiotherapy. For time calculations, completion of radiotherapy was used as starting point and death or last follow-up as an endpoint.

Correlation between expression percentages of our biomarkers of interest (HIF-1α, CA-IX, and Ki-67) was assessed using Spearman correlation. Also, relations between the expression parameters of the biomarkers, age, sex, clinical T-classification, and clinical N-classification were tested using the Mann–Whitney *U* test. Expression percentages of HIF-1α, CA-IX, and Ki-67 were dichotomised in accordance with previous studies of our institution [13, 14, 19] to facilitate comparisons with other studies.

Univariable and multivariable Cox regression analysis were performed to assess which variables were independently correlated with locoregional recurrence and disease-specific mortality. Besides our biomarkers of interest, the multivariable analysis contained a confounder set which was defined using a backwards stepwise model.

The proportional hazard assumption for Cox regression analysis was met as evaluated by LML plots. In all statistical tests, a *p*-value < 0.05 was considered statistically significant.

All statistical analyses were performed using SPSS (IBM Corp. Released 2015. IBM SPSS Statistics for Windows, Version 23.0. Armonk, NY: IBM Corp).

## Results

### Patients’ characteristics

From the 1669 patients with LSCC in our database, 61 met the inclusion criteria. Demographic and clinical data are described in Table 1. The cohort of 61 patients with advanced-stage LSCC consisted of mainly men (87%), with a median age of 69 years. Sixty-two percent had a T3 tumour, of which 74% had no regional lymph node metastases at the time of diagnoses. Median follow-up for all patients was 48.4 months (range 1.5–145.9), and 61.1 months (range 11.3–138.9) for all living patients. Thirty-three patients (54%) deceased during follow-up, 11 (18%) due to a consequence of LSCC. Sixteen patients (26%) developed a locoregional recurrence within 2 years after completion of radiotherapy, 11 without regional recurrent disease. No correlations were observed between the expression parameters of the biomarkers, age, sex, clinical T-classification, and clinical N-classification.

**Table 1:** Demographic and clinical data of patients (*n* = 61)

Characteristic	Number of patients (column %)	HIF-1α		CA-IX		Ki-67	
		Low	High	Low	High	Low	High
Total N	61 (100)	43 (70)^a^	18 (30)^a^	51 (84)^a^	10 (16)^a^	50 (82)^a^	11 (18)^a^
Age, years							
Median	69.1	68.0	71.6	65.3	76.5	70.1	61.0
Range	41.7–84.9	41.7–84.9	53.7–83.3	41.7–84.9	47.0–82.9	41.7–84.9	44.4–76.4
Age, years							
< 65	24 (39)	19 (79)	5 (21)	23 (96)	1 (4)	18 (75)	6 (25)
≥ 65	37 (61)	24 (65)	13 (35)	28 (76)	9 (24)	32 (87)	5 (14)
Sex							
Male	53 (87)	38 (72)	15 (28)	45 (85)	8 (15)	44 (83)	9 (17)
Female	8 (13)	5 (63)	3 (38)	6 (75)	2 (25)	6 (75)	2 (25)
Clinical T-classification							
T3	38 (62)	26 (68)	12 (32)	33 (87)	5 (13)	31 (82)	7 (18)
T4	23 (38)	17 (74)	6 (26)	18 (78)	5 (22)	19 (83)	4 (17)
Clinical N-classification							
N0	45 (74)	30 (67)	15 (33)	39 (87)	6 (13)	40 (89)	5 (11)
N1	10 (16)	8 (80)	2 (20)	8 (80)	2 (20)	8 (80)	2 (20)
N2	4 (7)	4 (100)	0 (0)	3 (75)	1 (25)	2 (50)	2 (50)
N3	2 (3)	1 (50)	1 (50)	1 (50)	1 (50)	0 (0)	2 (100)
Follow-up, months							
Median	48.4	57.7	17.5	48.4	45.1	58.0	29.9
Range	1.5–145.9	1.9–138.5	1.5–145.9	1.5–138.9	3.5–145.9	1.5–145.9	2.0–74.1

*N* number of patients

^a^Row % instead of column%

### HIF-1α expression is significantly associated with locoregional recurrence in advanced-stage LSCC

Patients with high expression of HIF-1α developed significantly more often a locoregional recurrence (39%) compared to patients with a low expression (21%) (*p* = 0.043) (Table 2). Although a significant correlation between the expression percentages of HIF-1α and CA-IX was found (*p* = 0.018), CA-IX and Ki-67 showed no association with locoregional recurrent disease (*p* = 0.710 and *p* = 0.077, respectively). Age, sex and T-status were not associated with locoregional recurrent disease. However, patients with regional lymph node metastases at diagnosis had a significantly higher chance of developing a locoregional recurrence compared to patients with an N0 neck at diagnosis (56% vs. 16%, respectively, *p* < 0.001) (Table 2). Multivariable Cox regression analysis revealed N-status as a strong independent predictor of recurrent disease (HR 11.74; 95% confidence interval 3.21–43.01; *p* < 0.001). And, interestingly, also HIF-1α was found as an independent predictor for the occurrence of locoregional recurrent disease (*p* = 0.001) (Table 2).

**Table 2:** Univariable and multivariable Cox regression analysis of immunohistochemical, demographic and clinical data in relation to locoregional recurrence

Characteristic	Univariable analysis			Multivariable analysis	
	Locoregional recurrence/number of patients (%)	HR (95% CI)	*p*-value	HR (95% CI)	*p*-value
HIF-1α					
Low	9/43 (21)	1	**0.043**	1	**0.001**
High	7/18 (39)	2.81 (1.03–7.61)		7.67 (2.21–26.62)	
CA-IX					
Low	13/51 (25)	1	0.710		
High	3/10 (30)	1.27 (0.36–4.46)			
Ki-67					
High	5/11 (45)	1	0.077		
Low	11/50 (22)	0.39 (0.13–1.11)			
Age, years					
< 65	8/24 (33)	1	0.346		
≥ 65	8/37 (22)	0.62 (0.23–1.66)			
Sex					
Female	3/8 (38)	1	0.490		
Male	13/53 (25)	0.64 (0.18–2.26)			
T-status					
T3	8/38 (21)	1	0.294		
T4	8/23 (35)	1.69 (0.63–4.51)			
N-status					
N0	7/45 (16)	1	**< 0.001**	1	**< 0.001**
N+	9/16 (56)	6.39 (2.35–17.36)		14.07 (4.10–48.34)	

Bold value indicates significant *p*-values (*p* < 0.05)

*HR* hazard ratio, *95% CI* 95% confidence interval

### Hypoxia and proliferation tumour markers are not associated with disease-specific mortality in advanced-stage LSCC

No significant relation was found between the expression of HIF-1α, CA-IX, and Ki-67 and disease-specific mortality (Table 3). Men had a significantly lower chance to die due to LSCC compared to women (13% vs. 50%, respectively, *p* = 0.021). In addition, patients with regional lymph node metastases at diagnosis had a higher chance of disease-specific death (50% vs. 7%, respectively, *p* < 0.001). Age and clinical T-classification were not related to disease-specific mortality. In multivariable Cox regression analysis, N-status was independently associated with disease-specific death (HR 10.28; 95% confidence interval 2.40–44.00; *p* = 0.002).

**Table 3:** Univariable and multivariable Cox regression analysis of immunohistochemical, demographic and clinical data in relation to disease-specific mortality

Characteristic	Univariable analysis			Multivariable analysis	
	Death due to LSCC/number of patients (%)	HR (95% CI)	*p*-value	HR (95% CI)	*p*-value
HIF-1α					
Low	8/43 (19)	1	0.783		
High	3/18 (17)	0.83 (0.22–3.14)			
CA-IX					
Low	8/51 (16)	1	0.324		
High	3/10 (30)	1.95 (0.52–7.37)			
Ki-67					
High	4/11 (36)	1	0.076		
Low	7/50 (14)	0.33 (0.10–1.12)			
Age, years					
< 65	5/24 (21)	1	0.760		
≥ 65	6/37 (16)	0.83 (0.25–2.73)			
Sex					
Female	4/8 (50)	1	**0.021**	1	0.104
Male	7/53 (13)	0.23 (0.07–0.80)		0.35 (0.10–1.24)	
T-status					
T3	6/38 (16)	1	0.659		
T4	5/23 (22)	1.31 (0.40–4.29)			
N-status					
N0	3/45 (7)	1	**< 0.001**	1	**< 0.001**
N+	8/16 (50)	11.25 (2.97–42.63)		9.73 (2.52–37.52)	

Bold value indicates significant *p*-values (*p* < 0.05)

*HR* hazard ratio, *95% CI* 95% confidence interval

## Discussion

This is the first study investigating the relationship between radiosensitivity determined by local control and tumour markers for hypoxia and proliferation in a homogenous group of patients with locally advanced (T3–T4, M0) laryngeal squamous cell carcinoma, treated with primary radiotherapy with curative intention. We only found an association between the expression of HIF-1α and locoregional recurrent disease. Hypoxia and proliferation (HIF-1α, CA-IX, and Ki-67) were not correlated with disease-specific mortality.

Literature data on the association between hypoxia markers and local or locoregional control after treatment with curatively intended primary radiotherapy showed controversial results [12]. Some studies found an association between local control after initial treatment with radiotherapy and CA-IX or HIF-1α in patients with laryngeal cancer [13, 21], while others could not confirm this [14, 22]. This might be explained by the diverse patient inclusion criteria, consisting of early-stage glottic carcinomas [13], early-stage supraglottic carcinomas [14], all early-stage laryngeal carcinomas [21], and a combination of advanced-stage laryngeal and hypopharyngeal squamous cell carcinomas [22]. Another explanation might be the different treatment regimens; in one study a large proportion of the included patients received concomitant systemic treatment (83%) [22], while the other studies only included patients treated with primary radiotherapy [13, 14, 21]. Therefore direct comparison of the present study with other studies is difficult because we investigated hypoxia-related tumour markers in a homogenous cohort of locally advanced-stage laryngeal cancers treated with primary radiotherapy without concomitant systemic treatment.

Hypoxia is crucial during tumour progression, as it leads to advanced vascularization and cell mobility and metastasis [23]. Because hypoxic tumour cells can be resistant to irradiation due to the requirement of oxygen for free radical formation to induce DNA strand breaks and cell death [24], many approaches to prevent hypoxia‐induced resistance have been examined [21]. HIF-1α is a transcription factor that was initially identified as an indirect marker of tumour hypoxia that responds to a decrease in oxygen levels within a cell [25]. In addition, the cellular response to hypoxia—promoting the transcription of CA-IX—is regulated by HIF-1α [26] in good agreement with the correlation between the expression of CA-IX and HIF-1α. Our small, however, homogeneous patient group combined with the possibly stronger prognostic and predictive abilities of HIF-1α compared to CA-IX [27] might explain that only HIF-1a was predictive for locoregional recurrence in our study. Ki-67 is a nuclear protein that binds antigens which are expressed in the active phases of the cell cycle (at G1, G2, M, mitosis) and are absent in resting cells (at G0). Therefore, Ki-67 is used as a marker for cell proliferation [28]. Ki-67 expression has been associated with clinical outcome in several studies, mainly in early-stage LSCC with discordant results regarding the association with locoregional control [12]. This is possibly due to the diversity of patient and tumour characteristics, and variations in immunostaining and scoring. To optimise the reproducibility, and minimise variable staining intensity and interobserver variability, we used standardised automatic immunostaining and automated digital scoring. Using automated Ki-67 staining and assessment, Ki-67 expression was not associated with response to radiotherapy. The lack of an association found in our current homogeneous group of patients with advanced-stage LSCC, is in agreement with our earlier findings in a series of early-stage LSCC [19].

The major strength of our study is the unique, homogenous patient group and the long follow-up time. However, due to the selection of a homogenous population of advanced (T3–T4, M0) laryngeal tumours treated with primary radiotherapy with the curative intention only, the number of patients included is small and, therefore, independent studies are needed to confirm our data.

There has been no improvement in the survival of advanced-stage laryngeal cancer over the last decades, possibly due to the shift to larynx-preserving treatment [29–31]. For decision-making regarding treatment policy, predicting radiosensitivity would be very useful with the clinical consequence of choosing surgery as the primary treatment in radioresistant tumours. This might improve survival, and the worse functional and oncological outcome of salvage therapy compared to primary surgery could thereby be avoided [11]. Therefore, the use of proliferation and hypoxia biomarkers is highly clinically relevant to optimize treatment decision-making.

Different approaches have been tried to overcome or reverse tumour hypoxia, for instance breathing hyperbaric oxygen or hypoxia-activated prodrugs and fluorocarbons [32]. However, due to limited efficacy and/or adverse effects these methods have proven to be insufficient. Currently, the safety, tolerability and effectiveness of other agents to overcome tumour hypoxia are being tested, for instance NVX-108, a nano-emulsion of dodecafluoropentane which increases tumour pO_2_ up to 400% and significantly improved radiosensitivity in radiated animals [33]. Hypoxia biomarkers could be used to identify patients who might benefit from these treatment modulations.

Tumour hypoxia also affects the microenvironment of the tumour, including tumour infiltrating lymphocytes (TILs) [34]. TILs are nowadays of high research interest in cancer research, as new drugs targeting the immune system are available, also for head and neck cancers [35]. Research focusing on the effect of a hypoxic state on the response to immunotherapy could further help in predicting the therapy effect.

## Conclusion

In our study HIF-1α is significantly related to locoregional control in patients with advanced-stage LSCC primarily treated with radiotherapy with curative intention. More research is needed to clarify the potential predictive role of HIF-1α in patients with advanced-stage LSCC in clinical practice. Our study implies that patients with high expression of HIF-1α might benefit from approaches to overcome or reverse tumour hypoxia to enhance radiosensitivity.

## Data Availability

Data is available on request.
